# Spontaneous spinal cord infarction secondary to embolism from an aortic aneurysm mimicking as cauda equina due to disc prolapse: a case report

**DOI:** 10.4076/1757-1626-2-7460

**Published:** 2009-06-12

**Authors:** Bassel El-Osta, Ali Ghoz, Vinay Kumar Singh, Elrasheid Saed, Murad Abdunabi

**Affiliations:** 1 Trauma & Orthopaedics Surgery, Luton and Dunstable Hospitals NHS Foundation TrustLewsey Road, Luton, LU4 0DZUK; 2 Trauma and Orthopaedics, Leeds General HospitalGreat George Street, West Yorkshire, LS1 3EXUK

## Abstract

Spinal “stroke” is an uncommon cause of paraplegia. Spinal cord infarction from unruptured aortic aneurysm is rare. When encountered it poses diagnostic challenge to the clinician due to its rarity, which may lead to incorrect or delayed diagnosis. We report a case of 62-year-old man presenting to casualty as caudaequina syndrome due to spinal cord infarction secondary to emboli from an infra renal abdominal aortic aneurysm. To the authors knowledge this is first case of its kind and has not been reported in literature. Patient had improvement in proximal motor function following repair of the aneurysm, although he remained doubly incontinent in six months follow up.

## Introduction

Spinal cord infarction is a rare clinical entity characterized by a sudden onset of paralysis, bowel and bladder dysfunction, and loss of pain and temperature perception, with preservation of proprioception and vibration sense [1]. Spinal cord infarction is much less frequent than cerebral infarction, accounting for only 1-2% of all strokes. The pathogenesis and natural history of spontaneous or nonsurgical spinal cord infarctions remain largely unknown [1]. Authors would like to report an extremely unusual case of Spinal stroke secondary to embolisation form unruptured aortic aneurysm in order to increase the awareness of the entity amongst clinicians.

## Case presentation

A 62-year-old Caucasian man, service engineer presented to accident and emergency with sudden onset of low back pain whilst lifting a washing machine at work. Pain radiated to groins and was associated with progressive bilateral weakness with numbness in the legs, with more marked symptoms on the left side. There were no cardiovascular symptoms, and the patient had normal observations. He was previously fit and well. He had no medications. He was a smoker for around 40 years and smoked twenty cigarettes a day. He was referred to the orthopedic team as a possible case of cauda equina syndrome secondary to a prolapsed intervertebral disc.

Neurological examination revealed profound motor deficit in the legs with Medical Research Council (MRC) grade 2/5 muscle power in the hip flexors and complete paralysis in all other groups distally. All reflexes including the plantar reflexes were absent, and sensation was altered throughout both legs. Sensory and motor functions at level T12 were preserved. Perianal tone and sensation were both markedly reduced and he was unable to feel the passage of a bladder catheter. His vital signs were stable with normal pulse and blood pressure. Complete cardiorespiratory examination was normal and all peripheral pulses were palpable. However, abdominal examination revealed a nontender pulsatile mass.

**Figure 1.**
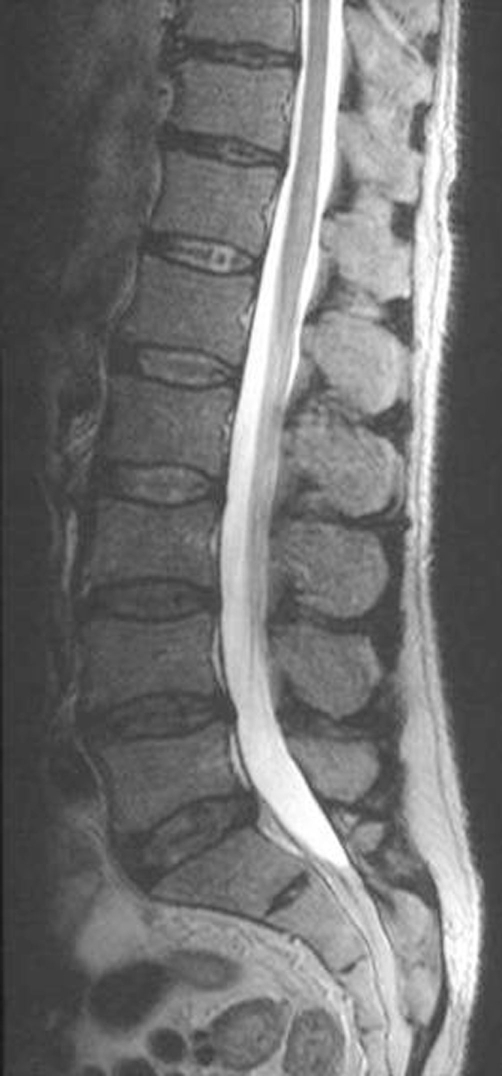
Saggital T2 weighted images showing multiple disc protrusion in lumbar spine.

**Figure 2.**
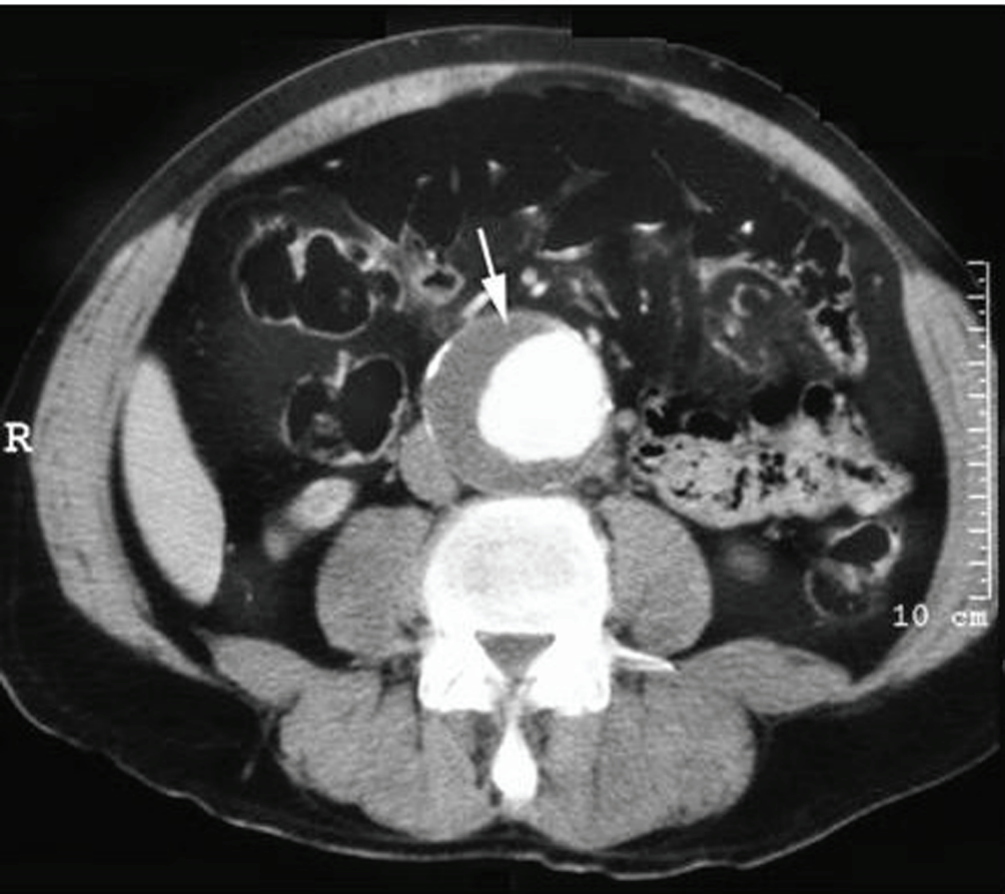
CT scan confirming 6.6 × 5.8 cm infrarenal aneurysm with thrombus in lumen.

Baseline laboratory investigations including Erythrocyte Sedimentation Rate were normal with the exception of a white cell count of 14.4 × 10^9^/Lt. Lumbar puncture was unremarkable. His other blood parameters including LFT, Renal functions and bone profile were normal apart from slight raised cholesterol 6.2(5.3 mmol/lt). Plain radiographs revealed mild degenerative changes in the spine. An urgent MRI scan was requested to rule out caudaequina due to disc prolapse. An MRI revealed (Figure 1) multiple disc protrusions in the lumbar spine, in particular at the L5/S1 level, but there was no evidence of acute cord or caudal compression. The scan also revealed the presence of an abdominal aortic aneurysm eroding the L3 vertebral body. An Ultra sound scan and a subsequent CT scan confirmed the presence of a 6.6 × 5.8 cm infra-renal aneurysm (Figure 2). Approximately, one third of the aortic lumen was filled by thrombus, but no leak or dissection was noted. A spinal arteriography was done early morning due to nonavailability of the investigation in out of hour's period. A diagnosis of spinal cord infarction secondary to emboli from an infra renal abdominal aortic aneurysm was made on the basis of all the above clinical, diagnostic and radiological tests.

Decision to repair the aneurysm wad made after multidisciplinary meeting to prevent further neurological deterioration secondary to further embolisation and to avert possible aneurysm rupture. Following the successful repair and an uneventful recovery, the patient was transferred to the regional spinal injuries unit for rehabilitation. At the point of transfer, he had MRC grade 3/5 muscle power in hip flexors, and grade 0/5 power in all muscle groups beyond this. He had a motor and sensory level of T12 and continued to have fecal and urinary incontinence, requiring bladder catheterization.

Follow up at 6 months showed an improvement in proximal motor function, with normal hip flexion bilaterally and normal knee extension on the right and MRC grade 3 on the left side. MRC grade remained 0/5 distal to the level of L3 bilaterally. Sensory level remained at T12, and whilst perianal sensation recovered, though he remained doubly incontinent. He was classed as having a neurological level of T12 ASIA “C”.

## Discussion

The spinal cord receives its blood supply mainly from the anterior spinal artery and the two posterior spinal arteries. Both run the length of the spinal cord and receive collateral supply. The posterior spinal arteries receive 12 unpaired radicular branches and the anterior spinal artery receives 7-10 unpaired radicular branches and hence has a less efficient collateral supply [1].

The anterior spinal artery is narrowest at the level of T8, the “watershed” area most liable to ischemia. The artery of the Adamkiewicz, the largest radicular artery usually arises at T9-L2 level and is on the left in 70% of the population [1].

Acute spinal cord ischemia is rare, accounting for approximately 5% to 8% of all acute myelopathies and 1% to 2% of all strokes [2]. The most prevalent etiology is atherosclerosis followed by aortic pathologies with or without surgery [3,4]. Other causes include degenerative spine disease, cardiac embolism, systemic hypotension, intercostals nerve block, and cryptogenic causes [5]. In our case, the patient was found to have an aortic pathology as the cause of his symptoms.

The rate of onset of paraplegia is one of the strongest indicators of the underlying pathology. A paraplegia or tetraplegia of sudden onset is most commonly due to injury. Typically, there is a history of trauma associated with this mode of onset. Occasionally the lesion is vascular or the result of acute myelitis [1]. With the latter, there is usually onset associated with fever, spinal tenderness, and root pain. Despite the rapid onset and severity of neurological deficit in our case, there was no history of trauma or any constitutional symptoms suggesting systemic infection, which has raised the suspicion of a vascular pathology [1].

Spinal MRI is an essential investigation in the diagnosis of spinal cord ischemia [6]. The main objective of MRI is to exclude other causes of acute cord compression [7]. This is extremely helpful in the diagnosis of other causes of paresis, which are more common than spinal cord ischemia. The second feature of MRI is T2 weighted images, which usually show hyperintense signal changes within the cord [6,7].

In our case, the management of this particular case consisted of conservative treatment and radical surgical approach towards the underlying cause by abdominal aortic aneurysm repair.

There seems to be some motor recovery since surgery and no further neurological deficit. We believe that the outcome at follow up is probably related to the initial motor deficit at presentation. Yet severe initial impairment (ASIA A and B) and female sex are considered to be independent predictors of unfavorable outcome [3]. The present case emphasizes the need of clinical suspicion and through abdominal and vascular examination in these cases for prompt diagnosis to avoid any associated patient morbidity and mortality [8].

## Conclusion

Spinal cord infarction secondary to abdominal aortic aneurysm is a rare presentation of acute nontraumatic paraplegia. Thorough abdominal and vascular examination in is imperative for timely diagnosis. Cord infarction is a well-known complication of dissecting aortic aneurysms and aortic surgery but can arise into hitherto asymptomatic and untreated abdominal aortic aneurysm. MRI remains the investigation of choice in the acute setting to rule out the most common causes.

## List of abbreviations

CT: Computed tomography
LFT: Liver function test
MRC: Medical Research Council
MRI: Magnetic Resonance Imaging
